# Assessing thermal changes in the equine thoracolumbar region following different capacitive-resistive electrical transfer protocols

**DOI:** 10.3389/fvets.2025.1570120

**Published:** 2025-05-21

**Authors:** Natalie Calle-González, Jose-Luis L. Rivero, Joaquín Olivares, Francisco Miró, David Argüelles, Francisco Requena, Ana Munoz

**Affiliations:** ^1^Department of Animal Medicine and Surgery, School of Veterinary Medicine, University of Córdoba, Córdoba, Spain; ^2^Equine Sports Medicine Center CEMEDE, School of Veterinary Medicine, University of Córdoba, Córdoba, Spain; ^3^Department of Comparative and Pathological Anatomy and Toxicology, School of Veterinary Medicine, University of Cordoba, Cordoba, Spain; ^4^Department of Electronic and Computer Engineering, Superior Polytechnic School of Córdoba, University of Córdoba, Córdoba, Spain; ^5^Department of Cellular Biology, Physiology and Immunology, School of Veterinary Medicine, University of Córdoba, Córdoba, Spain

**Keywords:** back, horse, radiofrequency, thermal therapeutic effects, thermography

## Abstract

**Introduction:**

Capacitive-resistive electrical transfer (CRET) is an endogenous non-invasive technique, used as deep diathermy. We pursue to analyze the temperature changes by applying different CRET protocols in the thoracolumbar spine of horses, between thoracic vertebrae 15 and lumbar 2.

**Methods:**

Ten clinically sound horses without thoracolumbar pain underwent various CRET protocols applied to a standardized thoracolumbar region (T15–L2). The protocols included sham (device off), low intensity (LIP, 5%), medium intensity (MIP, 30%), and high intensity (HIP, 40%). The HIP protocol was further divided into two-subprotocols based on the application of a subsequent low-intensity capacitive therapy: HIP+CAP (with capacitive therapy) and HIP-wCAP (without capacitive therapy). Skin minimum (Tmin), maximum (Tmax), and mean (Tmed) temperatures were assessed by thermography in degrees Celsius (°C) at assigned measurement times during application, and for 30 min post-therapy application.

**Results:**

No significant differences in Tmed and Tmax were found between sham and LIP protocols in any of the measurement times. During application, there were no significant differences between MIP and HIP protocols, but during the first 15 min after application, Tmed and Tmax were significantly higher in the HIP+CAP protocol (median and [interquartile ranges], 29.17°C [28.20–31.5°C]; 31.70°C [29.50–33.10°C]) compared to MIP (25.36°C [23.41–26.98°C]; *p* = 0.002; 27.58°C [26.15–28.10°C]; *p* = 0.001) and to HIP-wCAP (25.48°C [23.12–26.21°C]; *p* = 0.001; 28.22°C [27.10–29.21°C]; *p* = 0.004). At 30 min after CRET, Tmed and Tmax remained significantly higher in HIP+CAP (26.68°C [24.75–28.19°C]; 29.23°C [28.18–31.21°C]) compared to sham (23.16°C [22.11–25.23°C], *p* = 0.022; 25.15°C [23.12–27.10°C]; *p* = 0.001), and LIP (24.25°C [22.13–25.34°C], *p* = 0.023; 26.22°C [24.23–27.34°C]; *p* = 0.034).

**Main limitations:**

Skin temperature was measured, rather than using invasive techniques involving the insertion of thermal probes into muscles. Skin thickness and hair density may have affected temperature measurements.

**Conclusions:**

Low-intensity CRET induced similar temperatures compared to sham. Moderate and high-intensity protocols produced similar temperature increases; despite high-intensity sessions were limited to 10 min due to horse tolerance. Shorter high-intensity treatments may be easier to apply and adding a short time of low-intensity capacity therapy after high-intensity protocols, may help maintain elevated temperatures for longer periods of time, without significantly increasing the duration of therapy.

## 1 Introduction

Heat physical therapy is widely used to manage various physiological and pathological conditions in equine athletes. It supports recovery between training sessions, enhances performance during warm-ups, and is commonly used to treat musculoskeletal conditions such as muscle spasms, injuries, and pain, ultimately contributing to improved athletic performance.

The therapeutic effects of heat stimulate a range of thermophysiological responses, including increased tissue temperature and metabolism (1–3), vasodilation, improved local blood flow and oxygenation (4, 5), accelerated nerve conduction velocity (6, 7), enhanced tissue extensibility and muscle flexibility (8), and reduced pain and stiffness (9, 10).

Therapeutic heating modalities are generally categorized as either superficial or deep. Superficial modalities include massage (11), electromagnetic field (12), and hot packs or heat wraps (13, 14), that often have limited ability to heat deeper tissues effectively. In contrast, deep heating modalities like therapeutic ultrasound, including high-intensity focused ultrasound (HIFU) (15) and radiofrequency, can penetrate deeper tissue layers more efficiently. One such modality, Capacitive Resistive Electrical Transfer Therapy (CRET), is a form of deep diathermy that delivers energy and stimulates tissue metabolism via endothermic effects. These effects vary according to the intensity of application and the tissue impedance encountered during the electrical current transmission (16).

Back injuries in horses are a common source of pain, neuromuscular dysfunction, and reduced performance, significantly affecting their overall wellbeing (17–19). Such injuries can result in muscle atrophy specifically on the same side as the affected area (ipsilateral), particularly in the stabilizing multifidus muscles. This may be accompanied by compensatory overuse (hypertonicity), pain, and stiffness in the surrounding epaxial muscles, ultimately contributing to reduced spinal stability (20, 21). Previous studies suggest that CRET can effectively alleviate mild to moderate thoracolumbar and epaxial muscle pain and atrophy, with biomechanical improvements observed after four sessions of CRET over two consecutive weeks (21). These findings support the use of CRET as a valuable adjunct in equine rehabilitation and training regimens.

Understanding how tissue temperature responds to different CRET intensities across targeted anatomical regions is crucial for optimizing treatment plans. This study aims to evaluate the different temperature responses in a standardized thoracolumbar region (T15–L2) of clinically sound horses free of thoracolumbar pain, following varying CRET intensity protocols, contributing to more effective therapeutic strategies. Our hypotheses are as follows: (1) the application of a low-intensity CRET protocol will not significantly increase tissue temperature. Instead, temperature will increase progressively with the intensity of the therapy, while medium and high intensities will result in progressively greater thermal responses, with the highest values for the high-intensity protocol; (2) elevated temperatures will persist for at least 30 min post-treatment in medium and high intensity protocols; and (3) applying a low-intensity protocol following a high intensity session will promote a more rapid decrease in temperature, potentially mitigating excessive post-treatment hyperthermia.

## 2 Materials and methods

### 2.1 Animals

Ten healthy adult Spanish purebred horses (four mares and six geldings), aged 7–15 years and weighing 370 kg – 455 kg, were included over a 2-month data collection period. All horses were privately owned and remained non-sedated throughout the study. Inclusion criteria were the absence of thoracolumbar (mid to lower back) pain, as confirmed by digital palpation of the area and clinical gait assessment according to the American Association of Equine Practitioners (AAEP) guidelines. Horses with clinical lameness score of ≤ 2/5 on the AAEP scale were eligible. Locomotion symmetry was further evaluated using an inertial measurement unit (IMU) analysis system (Equigait Ltd., Chestnut, Herts, UK) compromising wireless sensors designed to detect and quantify vertical dorsoventral displacement during both the stance and suspension phases of either a forelimb or hindlimb, allowing for comparative analysis between contralateral limbs during trot (22). Specific thresholds were established to determine acceptable locomotor symmetry: a difference of < 12 mm in minimal head displacement (HDmin), defined as the difference in the lowest vertical position of the head during the stance phase of the left and right forelimbs, expressed in millimeters, and < 6 mm in minimal pelvic displacement (PDmin), which refers to the difference in the lowest vertical position of the pelvis during the stance phase of the left and right hindlimbs, measured in millimeters (23). Horses exceeding these thresholds were considered asymmetrical and excluded from the study.

### 2.2 Capacitive resistive electrical transfer device

The CRET treatment was applied using a monopolar radiofrequency device operating at a fixed frequency of 448 kHz (Animal Health Vet905, Indiba^®^ S.A.U, Barcelona, Spain) (24). Two types of active electrodes, capacitive (CAP) and resistive (RES), were used to deliver the therapy. These electrodes emitted high frequency electrical currents, which were transmitted through the horse's tissues and returned via a neutral or inactive electrode (a flexible, 200 mm x 260 mm metal plate) secured to the sternal area using an elastic strap.

Both active electrodes were used alternatively, not simultaneously, and are both metal made. The capacitive (CAP) electrode is coated with a polyamide layer, acting as a dielectric medium to prevent direct contact between the metal surface and the skin. This setup creates a capacitor with the treated tissues, producing localized electrical changes and heating superficial structures. In contrast, the resistive (RES) electrode is uncoated and not insulated, allowing deeper tissue penetration of the radiofrequency current. The active electrodes have sizes comprised between 35 and 65 mm in diameter. In our study, the size of both active electrodes was 65 mm in diameter, chosen due to the extended treated area (24).

### 2.3 Capacitive resistive electrical transfer protocols

Horses were randomly assigned to receive different CRET protocols, with treatment applied to either the left or right side (balanced 50/50). A 4 day (“washout period”) was left between each treatment protocol for each horse, to avoid carryover effects (25). The protocols included: (1) low intensity protocol (LIP); (2) medium intensity protocol (MIP); (3) high intensity protocol (HIP), further divided into two different sub-protocols, each performed on five animals: the HIP+CAP and HIP-wCAP protocols, with and without capacitive therapy after HIP, respectively; (4) sham protocol (sham), conducted with the device switched off, to control for the effect of electrode mechanical movement alone, determining if it induces changes in skin temperature, thereby contributing to the thermal effects observed during active treatment. The characteristics of each treatment protocol are described in Table 1.

**Table 1:** Characteristics of the capacitive resistive electrical transfer protocols evaluated in the study.

**Protocol**	**Total time of therapy**	**Capacitive therapy (CAP)**	**Resistive therapy (RES)**	**Intensity**	**Subprotocols**
sham	20 min	5 min	15 min	With the machine-off	No
LIP	20 min	5 min	15 min	5%, low intensity	No
MIP	20 min	5 min	15 min	30%, medium intensity	No
HIP	15 min	5 min	10 min^*^	40%, high intensity	HIP+CAP: 5% of low-intensity capacitive therapy after HIP HIP-wCAP: without low-intensity capacitive therapy after HIP

^*^Animals in this study could not tolerate the high intensity protocol (HIP) with the RES electrode for more than 12 min.

**Figure 1:**
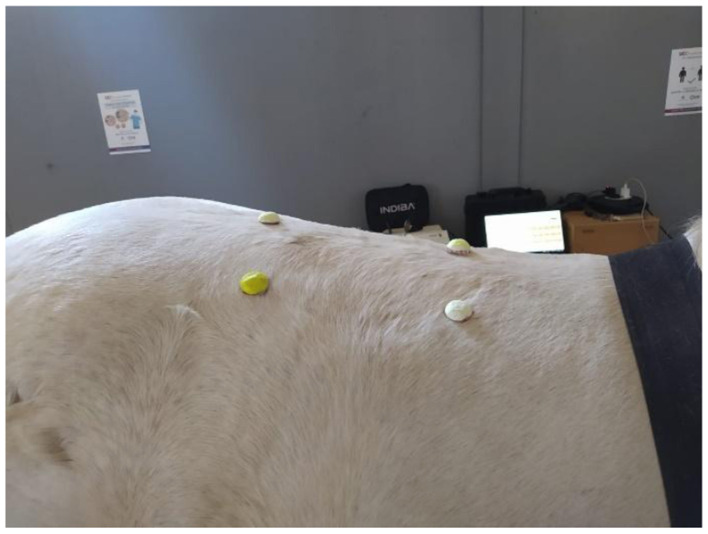
Standardized area of treatment, between the 15th thoracic vertebra and the 2nd lumbar vertebra (T15–L2).

In all cases, the treatment area was standardized as a rectangular region located between the 15th thoracic vertebra (T15) and the 2nd lumbar vertebra (L2), using T18 thoracic vertebra as a midpoint reference. The vertical height of the treated area was defined as half the distance length between T15 and L2. Cork marker dots attached with Velcro to the animal's skin were used to visually define the region (see Figure 1). Hair samples were taken to ensure consistency across subjects, with hair lengths ranging from 0.9 mm −1.2 mm. All horses included in the study were sport horses with short hair coats, housed individually in box stalls.

### 2.4 Thermographic temperature measurement

Prior to CRET therapy, horses were housed indoors in individual box stalls without direct sunlight for 30 to 40 min to stabilize their body surface temperatures, following established thermography protocols (26–28). Once acclimated, the designated treatment area was prepared and baseline thermographic measurements were obtained, followed by the application of a standardized amount of water (250 ml of water at 23°C) and gel (50 ml of gel at 11°C) before treatment. Meteorological data (ambient temperature and humidity) and rectal temperature were recorded for each horse before and 30 min after each session (see Table 2), to control for potential physiological fluctuations. The study was conducted during the fall season, from October 1st to December 1st, maintaining consistent weather conditions. All CRET protocols were performed under stable environmental conditions, at consistent times of day, by the same operator (N.C.G), and with the same equipment, to reduce variability. Uniform housing and handling procedures were maintained throughout the study, to control confounding factors.

**Table 2:** Median values and standard deviations of environmental and physiological parameters before and 30 min after the application of the four CRET protocols.

**Protocol**	**Timepoint**	**Median temp (°C)**	**SD Temp**	**Relative humidity (RH) (%)**	**SD RH**	**Rectal temp (°C)**	**SD Rectal temp**
SHAM	Before	18.4	0.276	36	0.0092	37.3	0.231
	30 min after	18.4	0.107	35	0.007	37.3	0.12
LIP	Before	18.0	0.201	37	0.0097	37.25	0.16
	30 min after	18.15	0.106	36	0.009	37.3	0.114
MIP	Before	18.25	0.179	37	0.0135	37.3	0.133
	30 min after	18.3	0.119	36	0.009	37.3	0.077
HIP	Before	18.2	0.17	36	0.0094	37.15	0.197
	30 min after	18.25	0.116	36	0.008	37.2	0.123

Thermographic images were taken using a FLIR model i7 thermographic camera (Teledyne FLIR, ES) with a thermal sensitivity (NETD) < 0.1°C at 25°C, FOL 7 mm lens, and a 140 × 140 pixel resolution (29). The camera was mounted on a tripod, positioned 1 m away from the treatment area, and placed vertically and perpendicularly to the measurement area. Images were taken in a closed room to eliminate air drafts and sunlight exposure.

**Figure 2:**
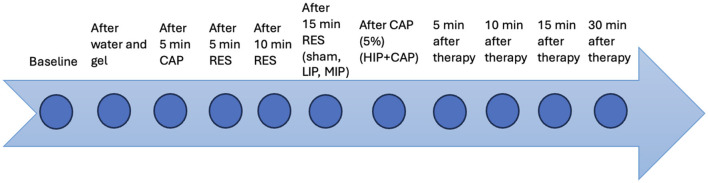
Thermographic measurement times after the application of the different protocols evaluated.

Three thermographic consecutive images were captured at the following times: baseline (before each protocol); after application of water and gel (w+g); after 5 min of CAP therapy (5minCAP) in all cases; after 5 and 10 min of RES therapy (5minRES, 10minRES) for all protocols; after 15 min of RES therapy (15minRES), in sham, LIP, and MIP only; after 2 min of CAP therapy (2minCAP), in HIP+CAP protocol only; and at 5, 10, 15 and 30 min after treatment. A representation of the thermographic measurement times is shown in Figure 2.

The 30-min post-treatment monitoring period was selected to assess the immediate thermal response to CRET. This timeframe allowed us to observe acute physiological effects while minimizing external variables such as movement, in line with ethical research practices and previous veterinary thermography studies (30–34).

### 2.5 Thermographic image processing

The captured thermographic images were stored for further processing. The best quality images were selected and converted to greyscale and temperature matrix using BFIC software. A mask was then generated to define the region of interest (ROI) based on the location of the thermal markers (beacons), and the maximum (Tmax), minimum (Tmin), and mean (Tmed) temperatures of the ROI were calculated (35–37). For temperature calculations, the beacons in the image were automatically detected by using a model based on convolutional neural networks (CNN) implemented by Keras/TensorFlow (35). This model was applied to 80% of the dataset, along with their augmented images versions generated by data augmentation. The model identified the beacons centers, and these were used to draw a quadrilateral outline of the ROI by using the Gift Wrapping algorithm (36).

Since this initial quadrilateral didn't fully account for the natural curvature of the horse's back, adjustments were made. Ambient temperature was estimated from the top 20 lines of the thermal images (focused always on the room), and the threshold mask was generated to refine the ROI. The final ROI included only points within the quadrilateral that exceeded the ambient temperature mask threshold, improving anatomical accuracy (37). Detailed image processing parameters are listed in Table 3.

**Table 3:** Generation of the mask to delimit the region of interest ROI, calculating the maximum (Tmax), minimum (Tmin), and mean (Tmed) temperatures of the ROI.

**Process for mask generation to delimit the ROI**	**Horse #1**	**Horse #2**	**Horse #3**
Thermal image.	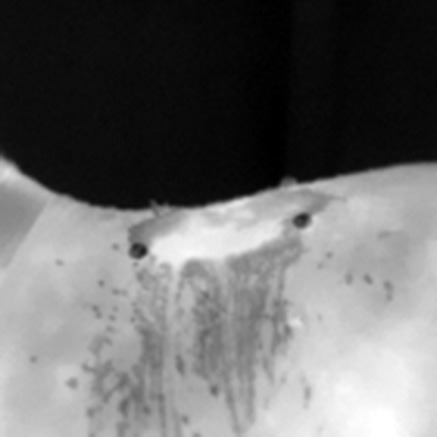	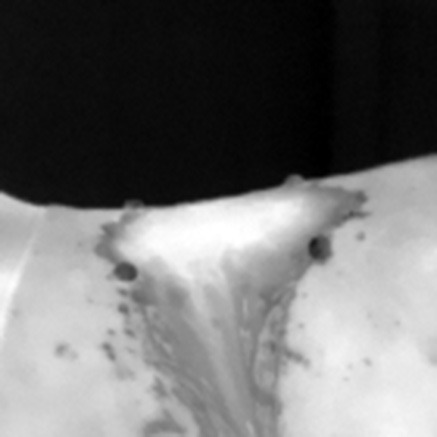	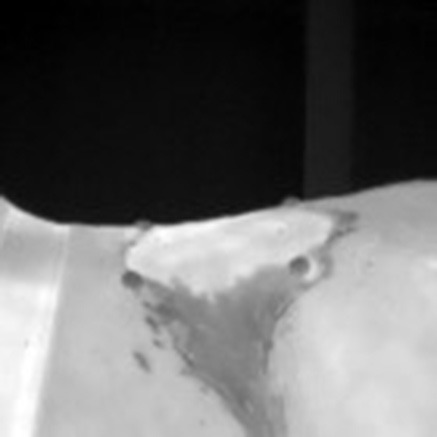
Beacons obtained by CNN.	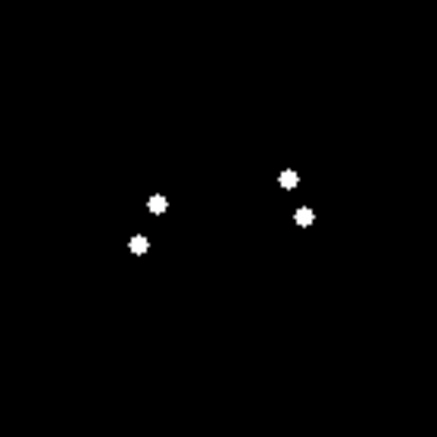	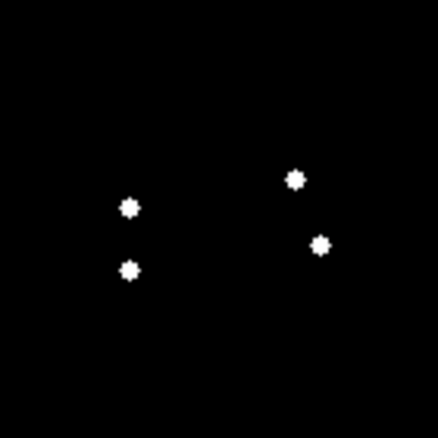	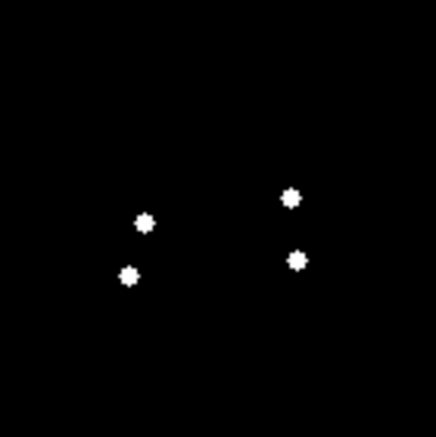
Polygon obtained from beacons.	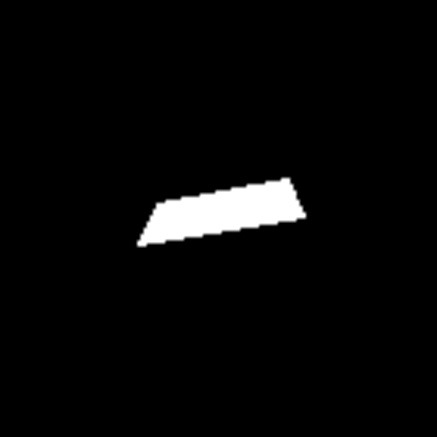	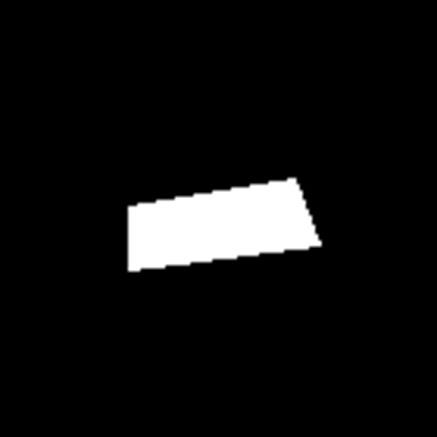	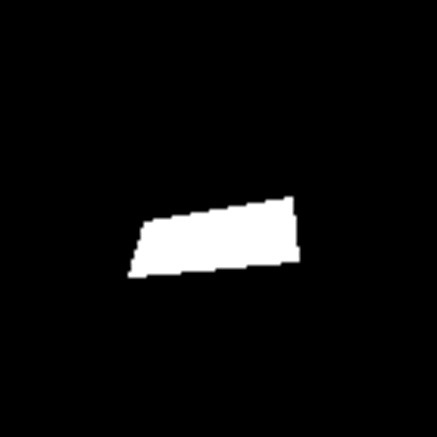
Environment temperature mask.	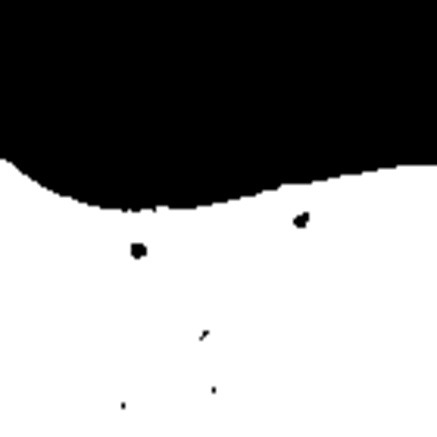	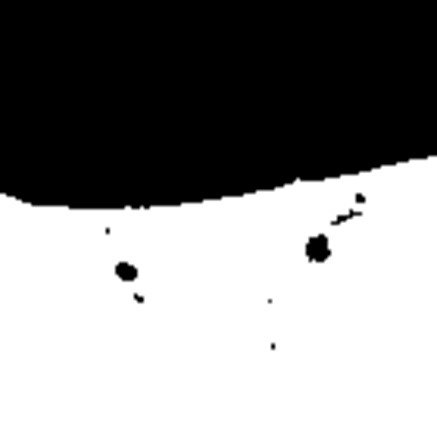	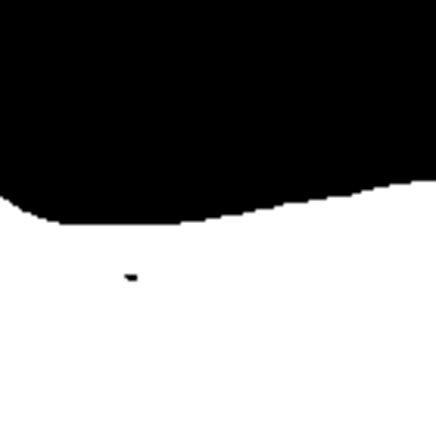
ROI: the intersection between the polygon and the environment temperature mask.	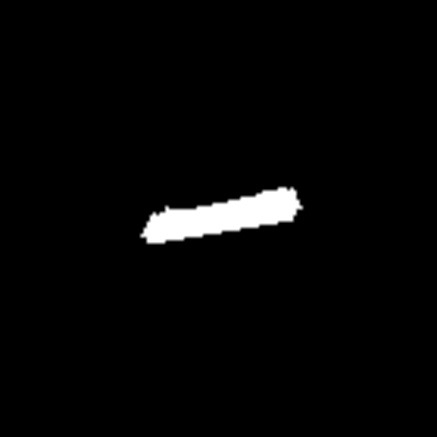	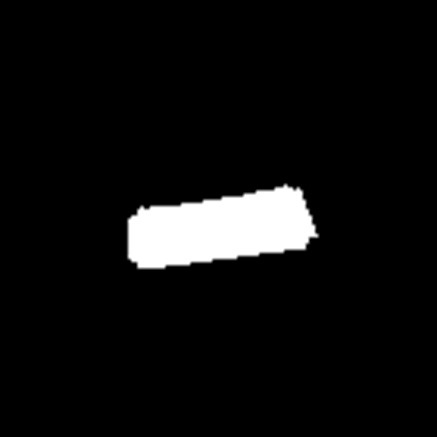	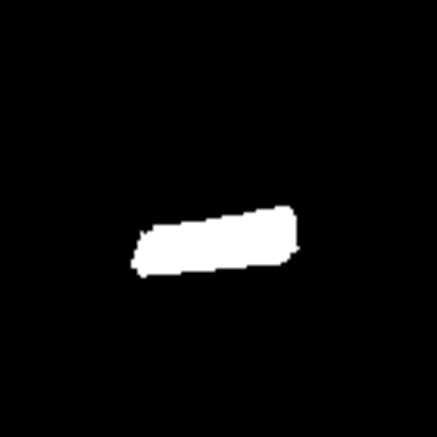
The ROI over the original thermal image.	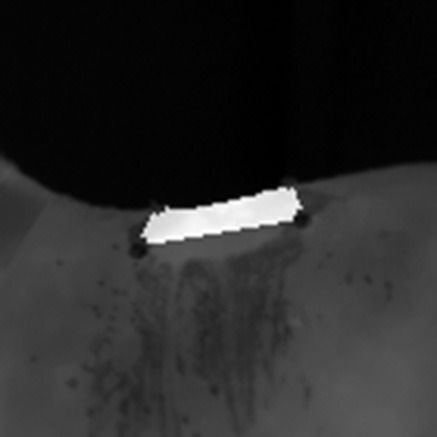	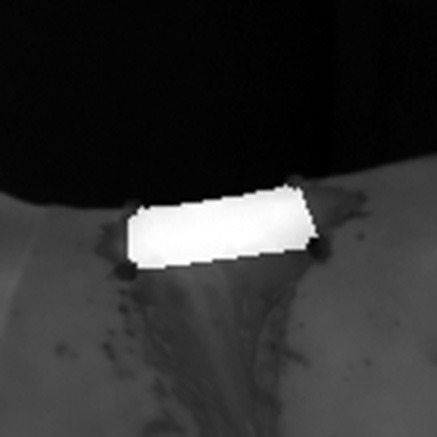	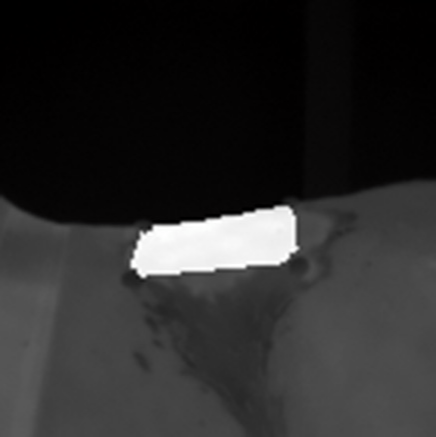

## 3 Statistical study

Data are presented as medians and quartiles. The normality of data was assessed using the Shapiro-Wilk test, and the homoscedasticity of variance using the Levene test. Data were not fitted to a normal distribution. Differences in Tmax, Tmin, and Tmed between each of the measurement times in each of the CRET application protocols and between protocols were assessed using Friedman's repeated measure test and a Kruskal-Wallis test, respectively. Wilcoxon range and Mann-Whitney tests were used as *post-hoc* when necessary.

The effect size was calculated as the non-parametric Cliff's delta (38), which represents the degree of distributional non-overlap between the Tmax obtained at each of the measurement times compared to those obtained at w+g for the five protocols evaluated (Table 3). The statistic ranges from −1 to +1, with the extremes indicating no overlap between the two distributions and zero indicating complete overlap. The sign of the effect size statistic is a function of the order assigned to the two Tmax evaluated; in our case, a positive Cliff's δ value was obtained when the Tmax at the time of measurement was higher than the Tmax measured at w+g. The thresholds for interpretation of Cliff's δ were δ < 0.147, negligible effect; 0.147 < δ < 0.330, small effect; 0.331 < δ < 0.474, medium effect; δ >0.474, large effect. Cliff δ values were calculated by the Cliff delta calculator available online (39).

In addition, ambient temperature, relative humidity, and rectal temperature before and after the therapies and between days were compared with a *t*-test for repeated measurements and a one-way ANOVA, respectively.

Statistical analysis was performed with IBM SPSS Statistics 21. Graphs were generated using Statistica for Windows (v. 13.0). In all cases, *p* < 0.05 was considered significant.

## 4 Results

All horses tolerated sham, low-intensity (LIP), and medium-intensity (MIP) CRET protocols without adverse effects. However, none could tolerate the high intensity protocol (HIP) with the RES electrode for more than 12 min. No significant side effects were observed, and environmental conditions and rectal temperatures remained consistent before and after therapy on all treatment days (see Table 2).

### 4.1 Differences between protocols

**Figure 3:**
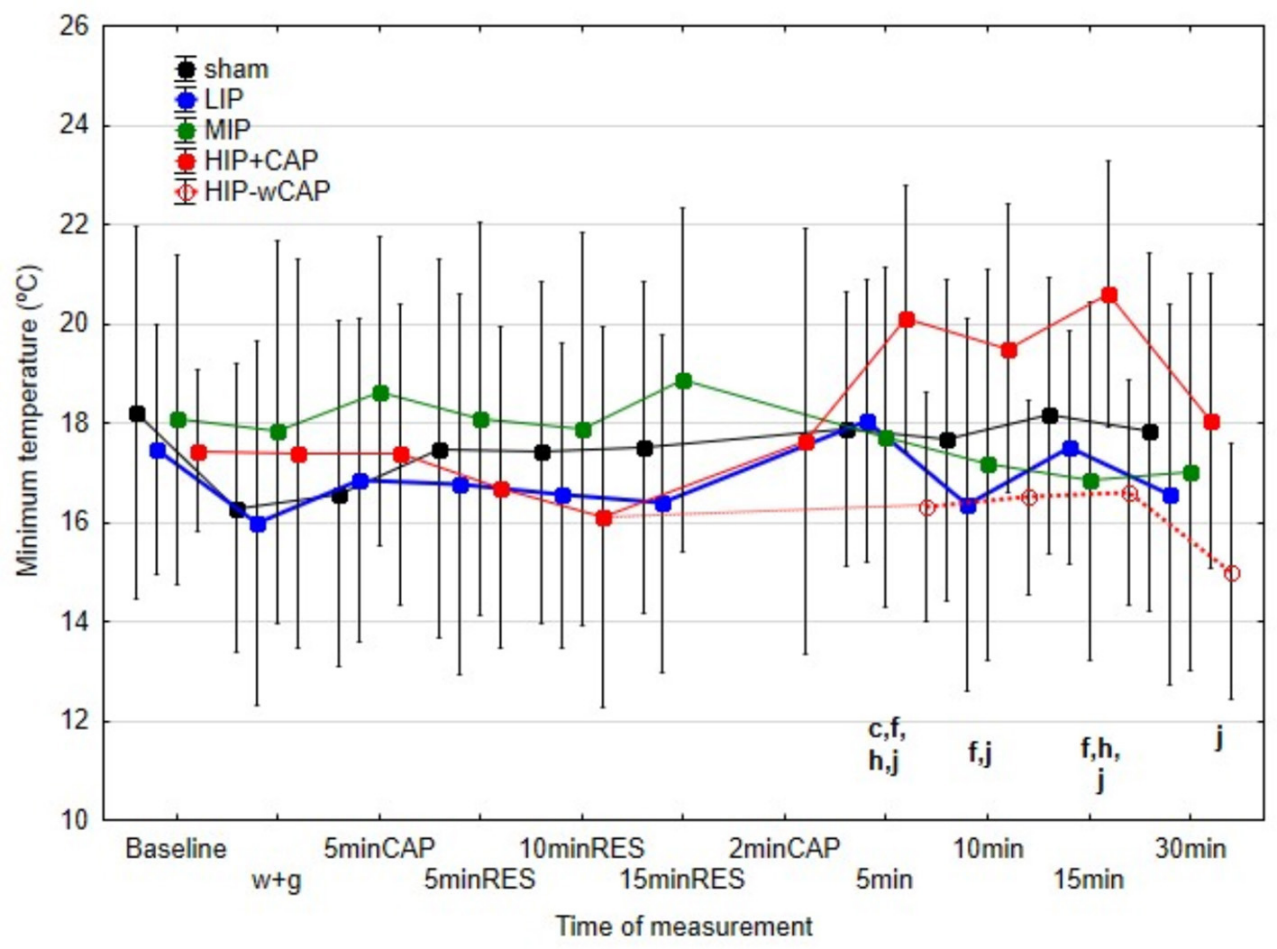
Median and interquartile range (IQR) of the minimum temperatures (°C) for all the protocols at the various measurement times (significant differences indicated by letters at *p* < 0.05); c: differences between sham and HIP+CAP; f: differences between LIP and HIP+CAP; h: differences between MIP and HIP+CAP; j: differences between HIP+CAP and HIP-wCAP).

**Figure 4:**
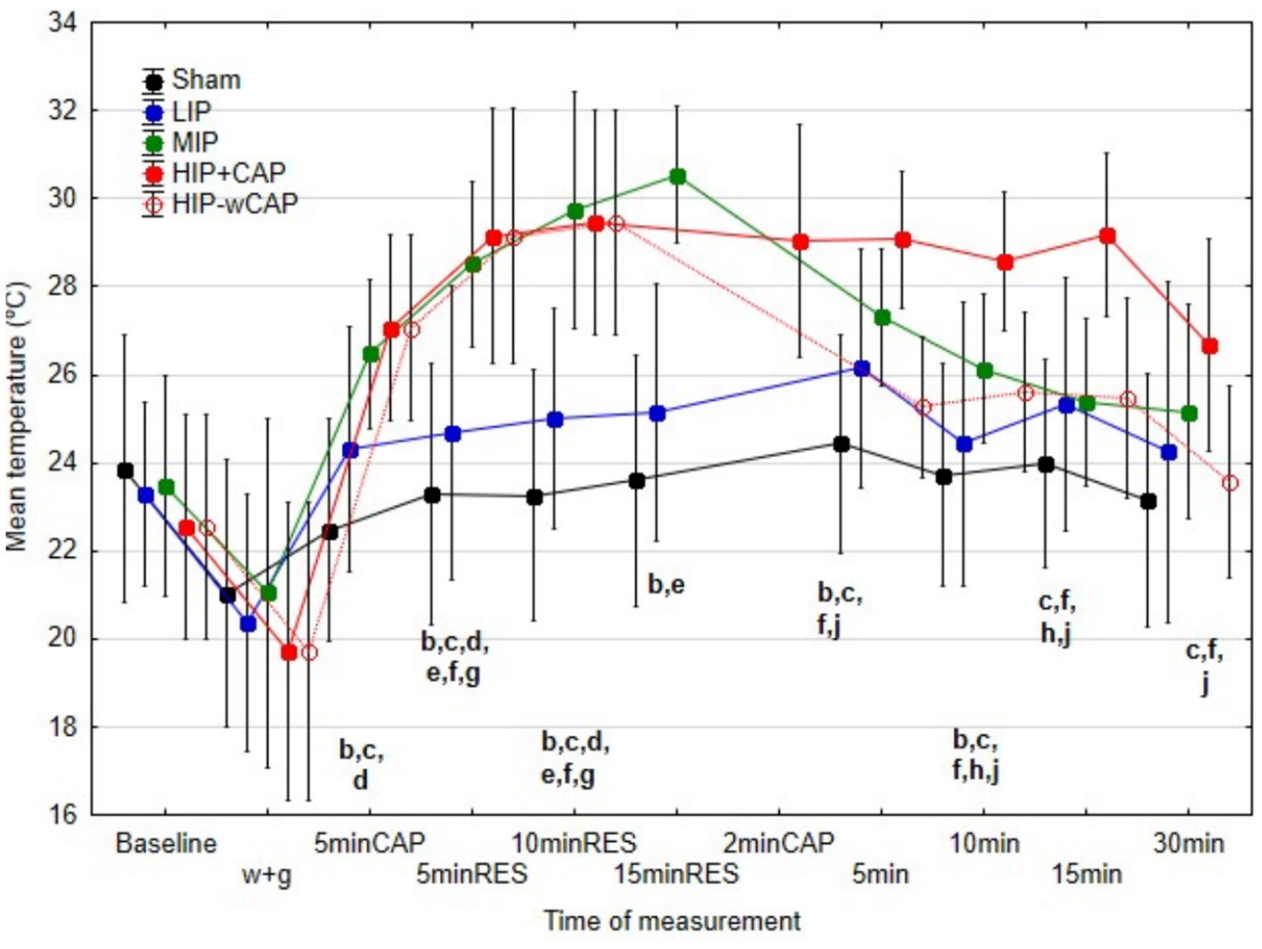
Median and interquartile range (IQR) of the mean temperatures (°C) for all the protocols at the various measurement times (significant differences indicated by letters at *p* < 0.05); b: differences between sham and MIP; c: differences between sham and HIP+CAP; d: differences between sham and HIP-wCAP; e: differences between LIP and MIP; f: differences between LIP and HIP-CAP; h: differences between MIP and HIP+CAP; j: differences between HIP+CAP and HIP-wCAP.

**Figure 5:**
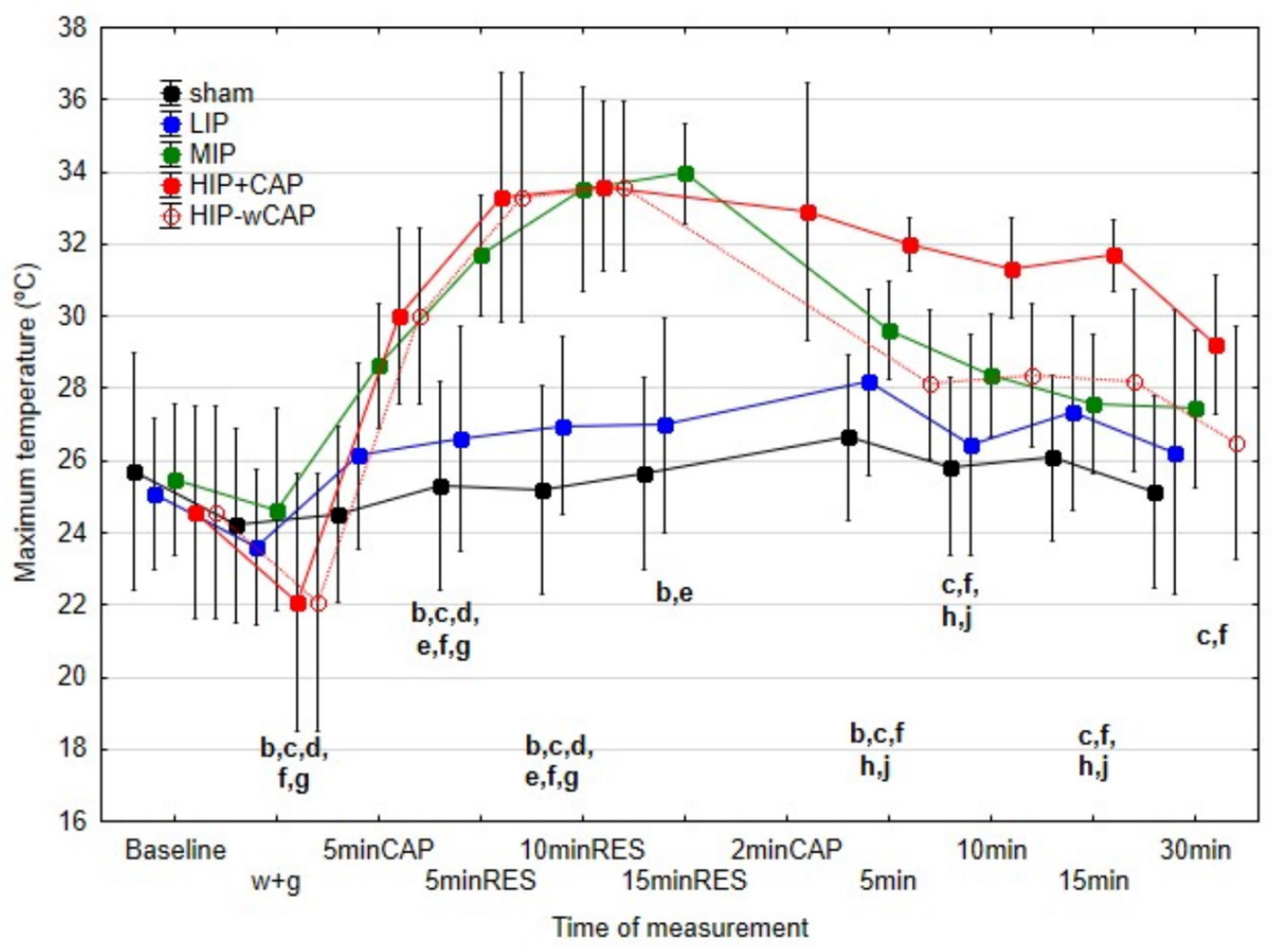
Median and interquartile range (IQR) of the maximum temperatures (°C) for all the protocols at the various measurement times (significant differences indicated by letters at *p* < 0.05); b: differences between sham and MIP; c: differences between sham and HIP+CAP; d: differences between sham and HIP-wCAP; e: differences between LIP and MIP; f: differences between LIP and HIP-CAP; g: differences between LIP and HIP-wCAP; h: differences between MIP and HIP+CAP; j: differences between HIP+CAP and HIP-wCAP.

Figures 3–5 illustrate differences in minimum (Tmin), mean (Tmed), and maximum (Tmax) skin temperatures across protocols.

#### 4.1.1 Minimum temperature (Tmin)

No significant differences in Tmin were found between protocols during therapy application. However, 30 min post-therapy differences revealed important contrasts. At 5 min post-therapy, only the HIP+CAP protocol produced significantly higher Tmin compared to the sham. By 15 min post-therapy, HIP + CAP Tmin remained significantly higher than in the MIP and HIP-wCAP protocols. At 30 min post-therapy, HIP+CAP protocol still exhibited elevated Tmin compared to HIP-wCAP (Figure 3).

#### 4.1.2 Mean and maximum temperatures (Tmed and Tmax)

There were no significant differences in Tmed and Tmax between sham and LIP protocols at any measurement time. During therapy, however, Tmed and Tmax were significantly higher in MIP, HIP+CAP, and HIP-wCAP protocols, although no significant differences emerged between these three protocols.

In post-therapy period, no significant differences in Tmed were observed among the sham, LIP, and HIP-wCAP protocols at 5 and 10 min. However, at 15 and 30 min, Tmed values for the HIP-wCAP protocol were significantly lower, 25.6°C and 23.5°C respectively, compared to the other four protocols, which remained statistically similar among themselves (Figure 4).

Tmax results in the post-therapy period, followed this pattern. At 5 and 10 min post-treatment, Tmax was significantly higher in MIP and HIP+CAP protocols compared to the other protocols. Notably, HIP+CAP protocol also exceeded MIP in Tmax. HIP-wCAP protocol showed no difference from MIP at these times. At 15 and 30 min post-therapy, HIP+CAP maintained significantly higher Tmax than all other protocols, with no significant differences between them (Figure 5).

#### 4.1.3 Absolute temperature changes

**Figure 6:**
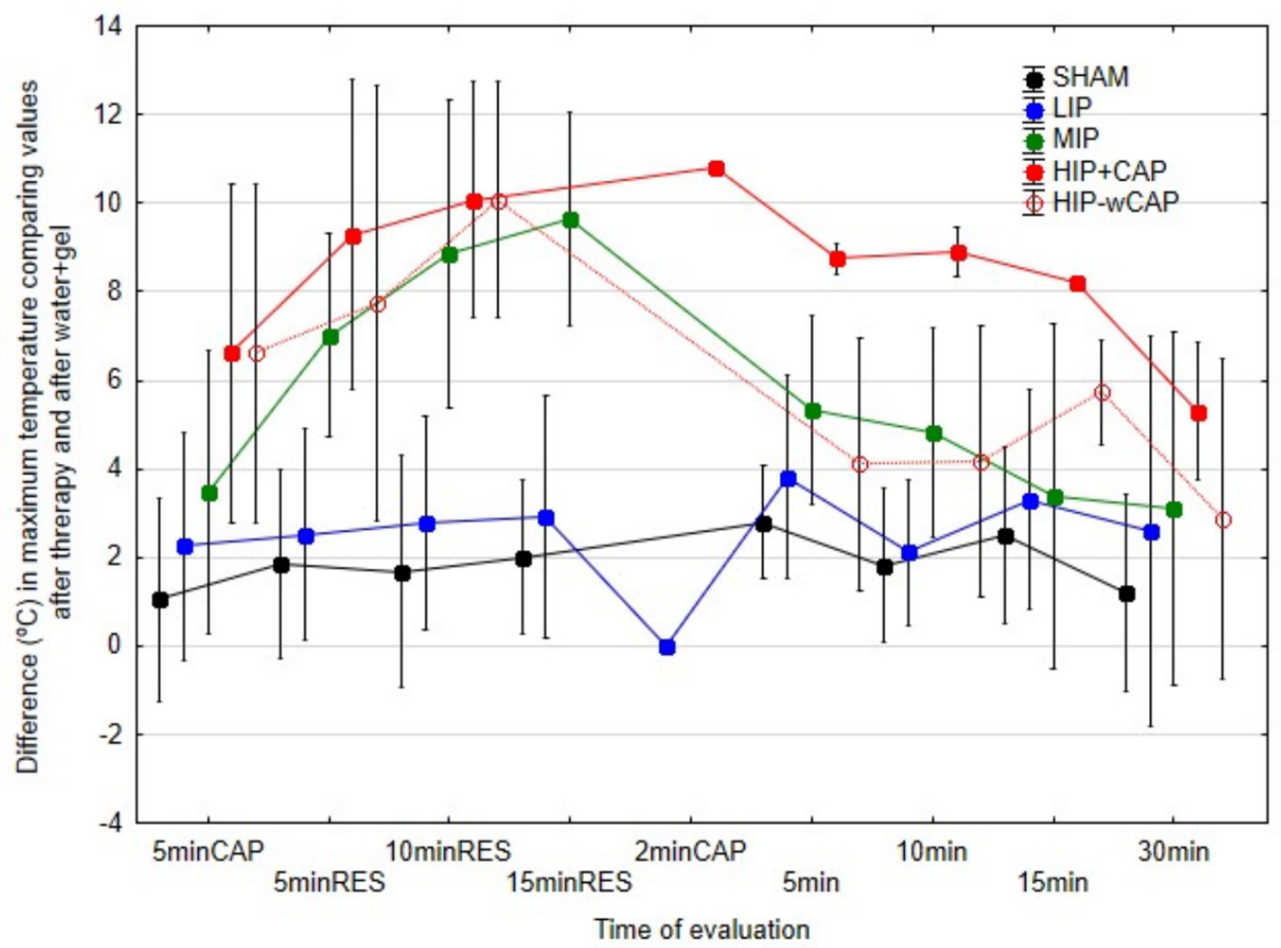
Differences in maximum temperature (°C) at each of the measurement times in relation to water + gel, in the various protocols.

To provide a practical view of the thermal impact, Figure 6 shows absolute temperature increases (i.e., Tmax at therapy end minus post water and gel (w+gel) application). MIP, HIP+CAP, and HIP-wCAP protocols induced the greatest increases in in Tmax at the end of therapy. A steep drop in Tmax was seen immediately after therapy in all protocols, except for HIP+CAP, which showed a slower decrease.

#### 4.1.4 Effect size (Cliff's δ)

The magnitude effect of Cliff's δ is presented in Table 4, further supporting these findings. Sham and LIP protocols showed small to medium effect sizes, while the most intense protocols, MIP, HIP+CAP, and HIP-wCAP generated large effect sizes during therapy application. Large effects were also observed at certain post-therapy time points across all protocols.

**Table 4:** Cliff's δ effect size.

**Measurement times**	**Sham**	**LIP**	**MIP**	**HIP+ CAP**	**HIP-wCAP**
5minCAP	0.326^†^	0.460^†^	0.792^§^	0.899^§^	0.899^§^
5minRES	0.464^‡^	0.887^§^	0.869^§^	0.905^§^	0.905^§^
10minRES	0.465^‡^	0.886^§^	0.889^§^	0.903^§^	0.903^§^
15minRES	0.899^§^	0.770^§^	0.889^§^		
2minCAP				0.949^§^	
5 min	0.892^§^	0.841^§^	0.887^§^	0.948^§^	0.913^§^
10 min	0.893^§^	0.844^§^	0.840^§^	0.949^§^	0.912^§^
15 min	0.912^§^	0.791^§^	0.752^§^	0.978^§^	0.731^§^
30 min	−0.444^‡^	0.415^‡^	0.730^§^	0.939^§^	0.645^§^

Effect size calculated as non-parametric Cliff's δ, comparing Tmax at each of the indicated measurement times, considering Tmax after water and gel application;^†^small effect;^‡^medium effect;^§^large effect.

### 4.2 Temperature changes over time within each treatment protocol

#### 4.2.1 Sham protocol

No significant changes in Tmin, Tmed or Tmax were observed at any of the measurement times, indicating no physiological thermal response.

#### 4.2.2 Low-intensity protocol (LIP)

LIP protocol led to significant increases in Tmed and Tmax at the four measurement times during therapy compared to the values obtained after w+g application. For instance, Tmax remained significantly elevated to 26.98°C at 15minRES (quartiles 26.0–27.7°C), compared to 23.61°C after w+g application (quartiles 22.1–23.9°C). By 30 min post-therapy, Tmax had slightly declined to 26.22°C (quartiles 24.6–27.10°C) but remained elevated. Tmin remained unaffected.

#### 4.2.3 Medium-intensity protocol (MIP)

Tmin remained stable throughout MIP therapy, without significant differences. However, Tmed increased significantly and progressively over the entire duration of therapy, from 21.04°C (quartiles 19.80–21.92°C) after w+g to 30.54°C (quartiles 29.20–32.3°C) at 15minRES. Tmax showed a rapid rise early in therapy, reaching 33.51° (quartiles 32.15–34.22°C) at 10minRES and plateauing at 33.97°C (quartiles, 32.15–34.10°C) by 15minRES. Both Tmed and Tmax were significantly higher than post w+g application values.

#### 4.2.4 High-intensity protocol (HIP and sub-protocols)

Unlike other protocols, HIP resulted in a significant post-treatment increase in Tmin compared to the values obtained after w+g application. Median Tmin rose from 17.40°C (quartiles 16.85–19.10°C) after w+gel application to values of 20.10°C, 19.10°C, and 20.60°C at 5, 10, and 15 min post-therapy, respectively.

Tmax at the end of therapy for HIP protocols reached 33.60°C (quartiles 31.25–34.22°C). In the HIP+CAP sub-protocol, Tmed and Tmax remained elevated at 15 min post-therapy, 28.58°C (quartiles 26.32–29.11°C) and 31.70°C (quartiles 29.12–31.92°C) respectively, with statistically similar values recorded during treatment (10minRES). Although these temperature values began to decline by 30 min post-therapy, they remained significantly high as those found at 5minCAP.

## 5 Discussion

Capacitive resistive electrical transfer (CRET) therapy, a 448 kHz radiofrequency technique, is known primarily for its endogenous diathermic effects, though *in vitro* studies have also shown electrical effects (40, 41). Compared to other electrophysical modalities, such as therapeutic ultrasound, CRET is proposed to facilitate deeper tissue energy transfer, due to the current flowing between the active electrode (CAP and RES) applied over the skin, to the inactive electrode or return plate.

Understanding how different CRET protocols produce different temperature changes can guide targeted applications depending on the anatomical site and clinical goals, as blood flow, tissue characteristics, and impedance vary significantly. Therefore, the aim of this study was to evaluate temperature variations following the application of different CRET treatment protocols to a defined region between T15 and L2 of the thoracolumbar spine of horses free of pain. To ensure consistency throughout the study, efforts were made to standardize all conditions, including animal handling, the use of identical equipment, and maintaining controlled environmental conditions, as suggested by Soroko and Howell (26).

The present study confirmed our first hypothesis, that the low-intensity protocol (LIP) had minimal thermal effects, with no significant differences in Tmed or Tmax between sham and LIP protocols during therapy application. The slight post-therapy increases in Tmax with the LIP protocol (2–2.5°C), compared to values obtained after w+g, was attributed to the mechanical effect of rubbing alone, as this effect was also found in the sham protocol. Despite limited heat buildup, this may still be beneficial in acute conditions, where mild temperature rises (1–2°C) are associated with positive physiological effects as increased cell membrane action potential and nerve conduction, improved tissue metabolism, enzymatic activity, oxygenation, and reduced edema (1, 42, 43). Notably, small-to medium Cliff's δ effect sizes observed in the sham and LIP protocols suggest a modest but potentially meaningful thermal response. Thus, the LIP protocol may be suitable for treating acute injuries where the avoidance of hyperthermia is desired.

Our first research hypothesis also proposed that the thermal response would increase with protocol intensity, resulting in the lowest temperature values in LIP and the highest in HIP. This hypothesis was only partially supported by our findings. While both Tmed and Tmax values progressively rose in the MIP protocol compared to LIP during therapy, the HIP protocol did not produce a further elevation in temperature beyond MIP. In the author's opinion, this plateau may be attributed to ongoing heat dissipation through vasodilation, a phenomenon previously described in studies on equine contrast therapy (13). Correspondingly, Haussler et al. (13), in their study on cold-heat contrast therapy in the lower limb of four horses, observed that tissue temperature decreased more rapidly during the cooling phase than it increased during the warming phase, suggesting that equine tissues may exhibit greater resistance to heat than to cold. While this may reduce burn risk, it could limit the ability to reach higher therapeutic temperatures when needed. Additionally, the MIP protocol lasted 20 min (5minCAP, 15minRES), while the HIP protocol was shortened (5minCAP, 10minRES) due to animal discomfort after 12–13 min of therapy, reducing the expected cumulative heat.

All protocols were applied with the same electrode size and by the same trained operator (N.C-G.), maintaining consistent speed and movement patterns. Investigating the impact of varying electrode sizes, movement patterns, or stationary electrode application, could help determine whether higher thermal loads can be safely achieved, such as for trigger point therapy, thereby enhancing the understanding and optimization of CRET protocols in veterinary practice. Moreover, large Cliff's δ effect sizes for the more intense protocols (MIP, HIP+CAP, and HIP-wCAP) shown in Table 4, were not only statistically significant but might also be therapeutically relevant. Increases in tissue temperature >3–4°C are known to enhance collagen extensibility, muscle contractile activity, and tissue biomechanical properties, improving recovery and rehabilitation outcomes (26).

The second proposed hypothesis of the study suggested that elevated temperatures would remain for at least 30 min after treatment with MIP and HIP protocols. However, this was only partially supported by the findings. Unexpectedly, no significant differences in Tmed and Tmax were observed at post-treatment time points between the MIP and HIP-wCAP protocols. These findings suggest that the main advantage of the HIP-wCAP protocol lies in its shorter treatment duration and efficiency, rather than in achieving higher temperature levels or prolonged post-therapy hyperthermia.

The third proposed hypothesis, that the application of a short, low-intensity CAP therapy (2 min), after a conventional (CAP+RES) in HIP-based protocols would promote heat dissipation and accelerate temperature reduction, which will be useful in those cases where temperatures exceed the therapeutic targets, was not supported by the results. Instead, adding of 2 min of low-intensity CAP prolonged the duration of elevated Tmed and Tmax during the post-therapy period. Notably, the HIP+CAP protocol was the only one in which Tmed and Tmax remained significantly elevated 30 min post-therapy compared with the values obtained in the w+g time. This indicates that HIP+CAP protocol may be beneficial in clinical scenarios requiring sustained thermal effects, such as in chronic conditions, muscle hypertonicity, or when increased elasticity/flexibility is the goal of the treatment. The mechanism behind this prolonged temperature elevation remains unclear, particularly given the low intensity of the CAP application, being unknown whether similar effects would be achieved if the same CAP addition was applied after MIP.

Furthermore, all evaluated protocols demonstrated a large Cliff's δ effect during the first 30 min post-treatment. This effect may have particular clinical relevance in HIP-based protocols. The HIP-wCAP protocol, with its shorter application time, may be advantageous in field conditions or in horses that are difficult to manage, while still achieving comparable temperature increases to MIP. Conversely, the HIP+CAP protocol may be preferred when sustained hyperthermia is the therapeutic objective, due to its prolonged post-treatment thermal effect.

One of the main limitations of this study was the limited sample size. The horses included in this study were privately owned, and participation required informed owner consent, which was often difficult to obtain, particularly due to the extended duration and complexity of the protocols and measurements involved. All animals were referred to the Veterinary Clinical Hospital—Equine Sports Medicine Center of the University of Cordoba for evaluation due to poor performance, though not related to musculoskeletal conditions; most were referred for other issues such as respiratory problems or for training/fitness evaluation. The thoracolumbar region is commonly subjected to mechanical stress from factors such as saddle fit and rider weight, and even well-managed horses often exhibit some level of discomfort or reactivity in this area (17, 44). As such, within this referred population of equine sport horses, identifying individuals completely free of thoracolumbar pain proved particularly challenging.

Data normality was assessed using the Shapiro-Wilk test, which confirmed non-normal distribution. Consequently, effect sizes were calculated using Cliff's delta (δ), a non-parametric and robust measure suitable for such data. Based on established conversion formulas, a medium effect size (δ ≈ 0.35) aligns with Cohen's *d* ≈ 0.75, which was used for power estimation. Assuming α = 0.05 and 80% power, a minimum of 10 subjects was considered sufficient to detect medium-to-large effects using non-parametric tests (e.g., Wilcoxon signed-rank, Friedman), which are clinically and physiologically relevant in this context (39). However, the sample size may be insufficient to detect smaller effects (δ < 0.3), such as subtle differences between protocols (i.e., HIP+CAP vs. HIP–wCAP or sham vs. LIP). These comparisons should therefore be interpreted with caution. Despite this, the sample size was deemed appropriate for the study's primary aims, balancing statistical justification with ethical and logistical considerations inherent to intensive repeated measures designs in large animal research. The findings should be viewed as preliminary and hypothesis-generating (45).

Another limitation of this study is that temperature measurements were limited to the skin surface rather than deep tissues. Although prior studies in a limited number of horses, ranging from 4 to 10, have successfully used implanted needle thermistors in tendons (13, 46) and epaxial muscles (46, 47), to assess thermal responses to modalities like therapeutic ultrasound and cryotherapy without adverse effects, such invasive methods were not practically feasible in our study, due to the use of privately owned horses. As a result, thermography was employed as a validated, non-invasive alternative to monitor thermal changes (26). While this method offers valuable insights into surface temperature dynamics, future studies using experimental horse populations may allow for invasive techniques to more accurately assess deep tissue thermal responses and treatment penetration. Nonetheless, cited literature (31, 33, 34, 48–50) supports the correlation of thermographic data with superficial muscle activity and blood flow, highlighting its relevance in evaluating physiological responses to exercise and therapy.

An important consideration is whether the tissue temperatures achieved with the MIP and HIP protocols are sufficient to elicit therapeutic effects. In human studies, according to Hing et al. (51) and Cochrane (52), effective thermal modalities typically involve skin temperatures ranging from 38 to 43°C. Also, Munari et al. (16) reported that an increase of ~1°C may alleviate mild inflammation, while increases of 2–3°C can reduce pain and muscle spasms, and a rise of 3–4°C has been associated with enhanced collagen extensibility, increased muscle contractility via enhanced ATPase enzyme activity, and altered tissue biomechanical properties. From this point of view, the average temperature increases observed in our study, with Tmed of 1–2°C with the LIP protocol, ~8°C with the MIP protocol, and 8–9°C with the HIP protocol (at therapy completion), suggest that clinically relevant thermal effects were likely achieved. However, it is important to note the anatomical differences: equine skin is considerably thicker than human skin, averaging around ~3.8 mm compared to ~0.6 mm in humans (53), which may influence heat transfer and tissue penetration.

Haussler et al. (13) reported tissue temperatures exceeding 40°C at different tissue depths in horses using an external contrast therapy device. In contrast, the mean Tmax recorded in our study was ~34°C, with maximum individual values reaching up to 37°C. Several factors may account for these differences, including the presence or absence of hair clipping, the environmental conditions, differences in the anatomical treatment areas, and the specific measurement techniques employed. To explore the potential influence of hair clipping, we conducted a preliminary pilot study on three horses, measuring baseline temperatures in the same thoracolumbar region (T15–L2) both with and without hair clipping. No significant differences were observed between the two conditions. Furthermore, the average ambient temperature in our study was 18.16°C (ranging between 17.80°C and 18.50°C), notably lower than the 25°C reported by Haussler's study, which likely contributed to the lower baseline temperatures observed in our study before w+g application [data not shown; 22–24°C for Tmed, compared to 32°C reported by Haussler et al. (13), pre-treatment].

Additionally, our treatment was applied to the thoracolumbar region, whereas Haussler et al. (13) focused in the metacarpal area, which differs in vascularization and potentially in tissue impedance. The choice of temperature measurement technique may also contribute to the observed differences. While Haussler et al. (13) utilized a skin-contact thermistor, we employed infrared thermography. Nevertheless, prior studies in humans have demonstrated strong agreement between thermographic and contact thermistors readings under controlled conditions, with an average bias of just 0.75°C (54). This minor variation is insufficient to explain the 8–10°C difference in baseline Tmed values between Haussler's study (13) and ours, suggesting that differences in anatomical location and environmental factors may significantly influence thermal outcomes.

## 6 Conclusions

Our findings indicate that the low-intensity CRET protocol does not significantly differ from the sham procedure in terms of temperature change. While both medium and high-intensity protocols generated comparable thermal responses, the high-intensity protocol was less well tolerated, with horses showing discomfort after 12–13 min of application. Therefore, high-intensity CRET may be appropriate in situations requiring shorter treatment durations, such as in animals that are difficult to handle or cannot tolerate extended restrain. Moreover, to prolong the post-therapy thermal effect, a brief (2 min) application of low-intensity capacitive therapy, well tolerated by animals, is recommended.

## Data Availability

The raw data supporting the conclusions of this article will be made available by the authors, without undue reservation.
